# Science, DEI and expanded thinking

**DOI:** 10.1177/14550725251325497

**Published:** 2025-03-17

**Authors:** Matilda Hellman

**Affiliations:** Department of Sociology, 225313 Uppsala University Department of Sociology, Uppsala, Sweden; and; 154260 University of Helsinki Faculty of Social Sciences, Helsinki, Finland

**Keywords:** DEI, science, trump second administration, expanded thinking, diversity

An ongoing collaboration project between the European Drug Agency (EUDA), Uppsala University and the International Society for Addiction Journal Editors (ISAJE) aims to ensure diversity and inclusion in addiction science production. With the aims of expanding viewpoints, epistemologies and language use in addiction science publishing, the project tackles issues such as the under-representation of the global south and the non-English speaking world, as well as the dominance of homogenized mainstream epistemologies (Hellman et al., 2020).

Openness to different possible perspectives is fundamental to critical thinking. This is reflected in high scientific standards, which not only bring us closer to a common understanding of a truthful, well-justified, and proven “reality”, but also require us to be open to questioning our assumptions and views. The closer we come to agreement on the characteristics of reality, the more we must be willing to engage in inward-directed, problematizing criticism.

Individual researchers must continuously scrutinize their own thinking, its sources, assumptions, possibilities and limitations. Good scientific conduct is characterized by humility in an awareness of the fact that we can think incorrectly and that we are able to problematize absolute answers. Expanded thinking, or so-called enlarged mentality, involves subjecting one's own thoughts and intellectual habits to examination and questioning.

## What is DEI?

In the beginning of 2025, diversity as a value is, perhaps more than ever, threatened in the free democratic world. In January 2025, newly appointed US president Donald Trump signed an executive order that mandates the termination of all diversity, equity and inclusion (DEI) programs within the federal government, citing them as discriminatory and wasteful (Exec. Order No. 14151, 2025). This has great impact on the science-making concerned with society and social issues. In this issue of *Nordic Studies on Alcohol and Drugs*, 29 editors of addiction journals have penned an editorial that outlines a strategy for us to deal with the US administrations new policies (Babor et al., 2025).

DEI is a socio-organizational framework that lumps together *diversity* (i.e., presence of difference and variety) with *equity* (i.e., ensuring fair treatment, access and opportunities) and *inclusion* (i.e., all individuals provided possibility to partake in society). It is particularly used for practices that are seen to foster a more inclusive and fair society generally speaking and environment such as workplaces, schools, and communities, more specifically. In the context of the USA, it has arisen as a response to historical and ongoing social and structural inequalities such as racial discrimination, which has left marginalized groups facing systemic barriers.

As the country grew more diverse through immigration and cultural change, there was a need for institutions to reflect and embrace the diversity and multiculturality in the workforce and beyond. Striving for better representation and equality was seen together with companies’ and organizations’ recognition of diversity fostering innovation, better decision-making and enhanced performance in a globalized economy (Prenzel et al., 2024; Allen et al., 2021). Social movements such as the Civil Rights Movement, Sans Papiers and Girls’ Right to Education have stressed equity and inclusion as ways of ensuring an end to discrimination and suppression.

What has come to be viewed as a politicized and ideological element of DEI is the cultural shift toward viewing it not only as strategic in certain situations, but also the launch of its core idea as a fundamental programmatic imperative to permeate all society and all situations. Some have advocated DEI-values as necessary for progress, whereas others view them as divisive or excessive.

## The termination of DEI

President Trump's executive order on the termination of DEI programs from 20 January 2025 (Exec. Order No. 14151, 2025) directed federal agencies to eliminate, within 60 days, DEI-related offices, positions and initiatives, including training, grants and contracts. The order emphasized that it stands for a shift toward merit-based employment and resource allocation, ensuring all government actions align with the principle of “equal dignity and respect”.

Consequences were immediate, also for the addiction science field: the Nature National Institutes of Health – NIH website removed information on diversity supplements, which supported early career researchers with funding and mentorship, and materials from the Office of Diversity, Equity and Inclusion. Additionally, details about the UNITE initiative, aimed at addressing structural racism in biomedical research, are now absent. The removal of these resources has raised concerns, particularly given the modest progress the scientific community has made toward diversifying the workforce (Kozlov, 2025).

At the time of writing this editorial, the Trump administration has halted research grant reviews, causing a freeze on 80% of the NIH's 47-billion-dollar budget, which funds research across the USA and globally. The NIH, under the Department of Health and Human Services, implemented a broad suspension of external communications, including advisory-committee meetings, affecting the scheduling and processing of research grants. The suspension of grant reviews has caused significant uncertainty, particularly for early career researchers, who may face delays in funding and career setbacks, such as jeopardizing job security and research milestones (Kozlov, 2025).

As part of a broader shift under the Trump administration, materials related to diversity, equity and inclusion programs at NIH, including efforts to address structural racism and support under-represented groups in science, have been removed from the NIH website.

The concept of “equal dignity and respect”, emphasized in Trump's executive order can, in a naive interpretation, be seen simply as an expression of a strong push to meet ideology with ideology: those who voted for Trump were tired of what they saw was a prioritizing the protection of the weaker as a value above all else. They wanted a more so-called meritocratic order in which the most suitable and best always wins. But is there any existing approach that fully incorporates this meritocracy ideal and has it worked so far? If the entire world constituted a recruitment base: who would get jobs if selections were purely based on this ideal? A system that never allows to consider a range of different perspectives does not materialize dignity and respect, and it is ill-equipped to describe and deal with both the external world and its internal affairs.

## In this issue

Flyger Holflod et al. (2025) present a scoping review on addiction problems and treatment in Greenland preceding the 2016 national treatment strategy. Bjønness et al. (2025) have studied health and social care workers’ perspectives on public gathering spaces for people using drugs. A study by Kievišienė (2025) provides insight into the ways in which Lithuanian social workers employ a combination of procedural and meta-competencies with field-specific characteristics in their work with alcohol use problems. In an analysis of cross-sectional population studies, Grönroos et al. (2025) have identified trends in attitudes towards gambling among Finnish women and men. Forsström et al. (2025) have looked into concerned significant others to individuals that experience gambling associated with risk that seek support at a national gambling helpline.
